# Information interventions can increase technology adoption through information network restructuring

**DOI:** 10.1016/j.isci.2022.104794

**Published:** 2022-07-20

**Authors:** D. Cale Reeves, Matthew Haley, Amara Uyanna, Varun Rai

**Affiliations:** 1 LBJ School of Public Affairs, The University of Texas at Austin, Austin TX, USA; 2 School of Public Policy, Georgia Institute of Technology, Atlanta GA, USA; 3 Department of Mechanical Engineering, The University of Texas at Austin, Austin TX, USA

**Keywords:** Applied sciences, Green engineering, Solar terrestrial physics

## Abstract

The rapid adoption of residential solar photovoltaic (PV) is recasting the role of individual households as a dynamic and potent construct critical for emissions mitigation and resilience of the electricity system. As residential PV enters more risk-averse customer segments, broader deployment of residential PV depends on overcoming both financial and informational barriers to adoption. Fast-changing residential PV technologies and associated policies means there is both lack of information and often misinformation among customers—gaps that are addressed effectively with local, trusted information networks, especially for big-ticket items such as residential PV. Here, we use an extensively validated agent-based model of residential PV adoption to analyze the effectiveness of different information intervention designs in spurring PV diffusion. We show that intervention designs are effective when they balance long-distance connections and local reinforcement, matching the intervention to both the informational needs of potential adopters and the structure of the underlying network.

## Highlights

• Introduces a framework for comparing economic and informational intervention programs
• Demonstrates empirically validated agent-based model as an experimental laboratory
• Shows how new social ties can balance access to novel information with reinforcement
• Estimates returns to programmatic effort for informational vs. economic interventions

## Graphical abstract

**Figure:**
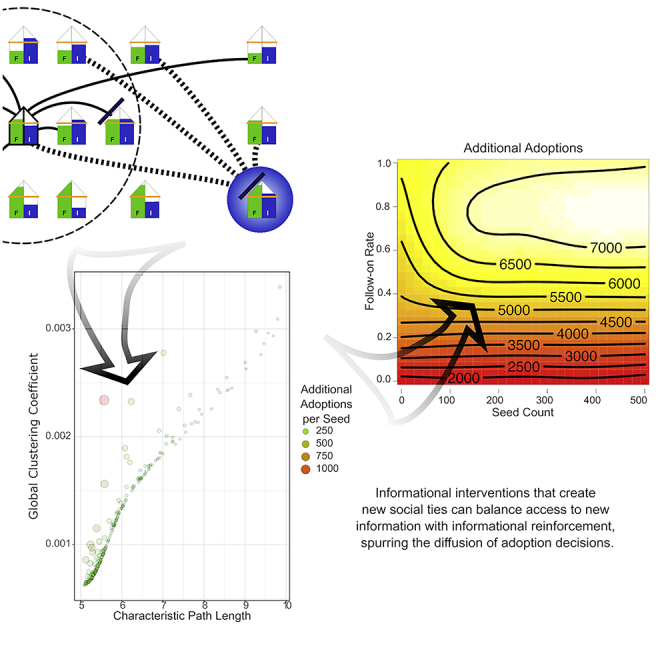

## Introduction

In addition to financial barriers, individuals face informational barriers to the adoption of new, green technologies. For residential solar energy systems, a variety of informational intervention programs (IIPs) have been deployed to increase diffusion. Here, we distill a variety of real-world IIPs into four archetypes, simulate their implementation using an extensively validated “virtual laboratory,” and assess their impact on diffusion.

We find that the mechanism for connecting informational “seeds” to those that receive information from them is an important determinant of IIP effectiveness. Against the backdrop of information exchanges along a small-world network, we show how an effective IIP balances two drivers: spreading information widely to reach many decision-makers and fostering the local reinforcement necessary to spur them into action. Our findings generalize beyond the empirical context presented here through structural similarities in underlying information networks.

**Figure 1:**
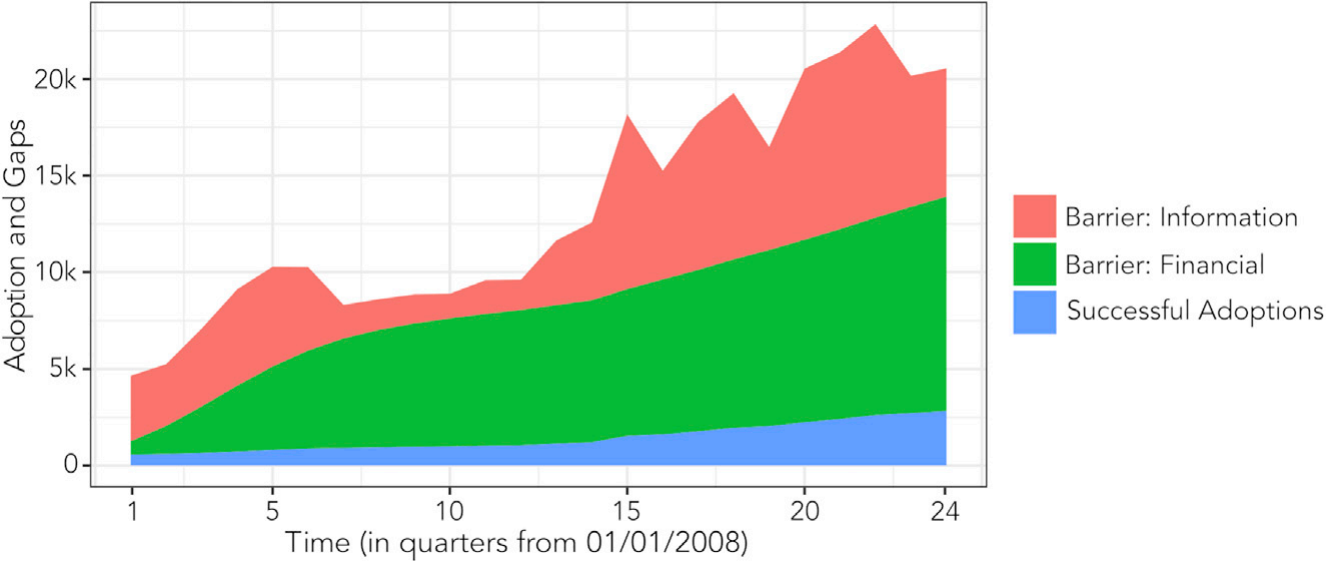
Illustrating the scale of informational barriers to residential PV adoption The solar PV adoption decision—driven by access to financial and informational resources—is successful when individuals can both afford the technology and have sufficient information to make the decision (shown in blue). A substantial proportion of the non-adopting population may still be able to afford the technology, but face an informational barrier to adoption (shown in red). Information intervention programs aim to supply individuals with the information needed to overcome these barriers. The data are from the validated model run without any simulated intervention; i.e. the baseline.

The management of energy supply and demand at the household level will play a central role in mitigating emissions of local and global pollutants from the residential sector (Goldstein et al., 2020; He et al., 2020), responsible for more than 20% of final energy consumption globally (IEA, 2020), and in the development of a smart, distributed energy infrastructure for making the electricity system more resilient and robust to natural disasters such as wildfires and hurricanes (Driesen and Katiraei, 2008; O’Brien and Hope, 2010; Yadoo and Cruickshank, 2012). Distributed generation, such as rooftop solar photovoltaics (PV), contributes to reducing the demand for centrally provided energy and, when the mix of fuels used to supply energy to the grid generates *C O*_2_ emissions, provides a decarbonized generation alternative. However, the rapid and intensifying proliferation of novel distributed consumer energy technologies including rooftop solar PV and electric vehicles (EVs) is giving rise to a new class of energy users—prosumers that both consume and produce energy services (Parag and Sovacool, 2016)—requiring a fundamental rethink of households as an active, dynamic, and more central part of the energy system (Sorrell, 2015; Rai and Henry, 2016). Although most policies and programs targeted toward accommodating the new realities of the energy system have focused on financial instruments—which have mixed cost-effectiveness (Borenstein, 2015) and may exacerbate equity issues (Barbose et al., 2018; O’Shaughnessy et al., 2021)—levers to unlocking prosumer potential operate in a much richer, intertwined behavioral and social context, best represented and understood as an emergent, complex system (Rai and Henry, 2016; Rai and Robinson, 2015). Viewed from this perspective, the heterogeneity of individual information sets and the dynamics of information exchanges are central to driving the patterns of consumer energy technology adoption and use, beyond purely financial drivers (Dietz et al., 2009, 2013; Allcott and Mullainathan, 2010; Robinson and Rai, 2015; Wolske et al., 2017). Individuals that do not have the information necessary for them to make a technology adoption decision face an informational barrier, and the scale of informational barriers to widespread diffusion can be substantial (see Figure 1). The dominant paradigm is shifting from promoting the rapid decarbonization of the residential energy sector through the adoption of home energy technologies to steering the evolution of a decarbonizing grid composed of prosumers. Efficient, cost-effective, and equitable strategies to support this transition while managing nuanced energy issues that arise at the individual or household level (e.g. geospatial disparities, socio-economic disparities, and so forth) will likely need full access to—and a full understanding of—a wider range of policy tools than financial instruments alone.

An important aspect of consumer energy technology adoption is that new information (e.g., about technology costs, performance, and reliable installers) not only needs to permeate through the system but also needs to be locally reinforced—that is, decision-makers reap the benefits of accessing new information when they are exposed to it from multiple sources—in order to spur change (Bollinger and Gillingham, 2012; Rai and Robinson, 2015; Graziano and Gillingham, 2015; Rai and Henry, 2016; Palm, 2016; Wolske et al., 2017; Barton-Henry et al., 2021). But these two aspects—long-range information proliferation and local reinforcement—do not always operate in sync over social networks and often entail non-trivial tradeoffs (Centola and Macy, 2007). In this article, we ask how information intervention programs (IIPs) can complement financial instruments and evaluate the comparative effectiveness of different IIPs to spark adoption decision-making. For the empirical test bed of our analysis, we use residential solar PV, which is increasingly being understood as the centerpiece of an evolving, co-adoption system of complementary energy technologies and behaviors that comprise distributed residential generation and consumption (Rai et al., 2016; Reeves and Rai, 2018; Sorrell, 2015).

IIPs have diverse design elements and often vary greatly from market to market (Costanzo et al., 1986; Palm, 2016; Sinclair and Rosoff, 2009). For instance, Table 1 highlights the variation in IIP design elements found in nine real-world IIPs in solar PV. IIPs in residential solar PV aim to address the informational needs of potential adopters directly and are often administered by solar community organizations (SCOs)—formal or informal organizations that provide potential adopters relevant information about solar PV and actively promote its adoption (Noll et al., 2014). However, by altering the levels and distribution of information in the decision-making context IIPs also have indirect effects through localized exchanges of information (Rai and Robinson, 2013; Rai et al., 2016). These so-called “ripple effects” (Hogan et al., 2004) contribute substantially to the effectiveness of informational interventions, potentially firing up adoption cascades, wherein a small subset of influential individuals can shift the attitudes of the masses and establish new norms that are powerful drivers of behavior change (Stoneman and David, 1986; Jachimowicz et al., 2018; Kovacs and Barabasi, 2015; Aral and Walker, 2012; Aral et al., 2013). The sustainable management of complex socio-technical-economic systems fundamentally depends on our understanding of how the interactive dynamics of policy and behavior lead to system-level outcomes (Castilla-Rho et al., 2017; Shove, 2010). However, the underlying complexity that propagates such effects also hinders systematically analyzing and comparing the effectiveness of different informational intervention programs. To address this challenge, we present a framework that enables the comparison of interventions with both economic and informational design elements.

**Table 1:** Real-world Informational Intervention Programs have substantial variation in implementation

Mass market advertising	Utility mailers, pamphlets, radio/TV ads, and call centers can reach a large number of potential adopters (Costanzo et al., 1986), but have been shown to be relatively less influential than neighborhood peer effects (Palm, 2016) and less frequently “spark” new interest in adopting PV than information exchanged through neighborhood peer-effects (Rai et al., 2016).
Open houses and guided tours	Local and national-scale solar tours engage peer effects by giving potential solar adopters the opportunity to interact with existing adopters (Palm, 2016; Noll et al., 2014).
Education and school events	Various organizations, including Solar Oregon, Pacific Gas and Electric (PGE), and other utilities have installed PV in public schools and designed solar-based curriculum training packages for teachers that fit state engineering and science benchmarks (Sinclair and Rosoff, 2009).
Community champion	These programs recruit members of the public from neighborhoods in their service area and provide them with technical training that enables them to coordinate and lead solar programs in their own communities (Noll et al., 2014).
Porch talks	Community solar advocates sitting on their porch after dinner and engaging with passers-by was an effective way to promote solar PV diffusion in the Mueller Megawatt Project (Noll et al., 2014).
Celebrity advocates	Advocates make endorsements designed to increase customer knowledge and awareness of a product (Kowalska-Pyzalska, 2016; Sinclair and Rosoff, 2009).
Local seminars, happy hours, and workshops	These programs hosted by SCOs educate potential adopters about the benefits of solar and have been influential in promoting solar PV adoption (Palm, 2016; Noll et al., 2014).
Group competitions	Organized between municipalities or between companies, these IIPs aim to encourage sign-ups to green power programs and to enlist employees as new solar customers (Sinclair and Rosoff, 2009).
Phone ambassadors	A website-hosted database connected existing solar adopters willing to share key information with prospective solar adopters (Noll et al., 2014).

Note that this list is neither meant to be exhaustive nor does it represent all IIPs that have been deployed in this empirical context. Rather, it is meant to highlight the variety of IIPs that are captured by the subsequently presented **mechanism**: **seeds**–**follow-ons** framework.

## The mechanism: seeds—follow-on framework

**Figure 2:**
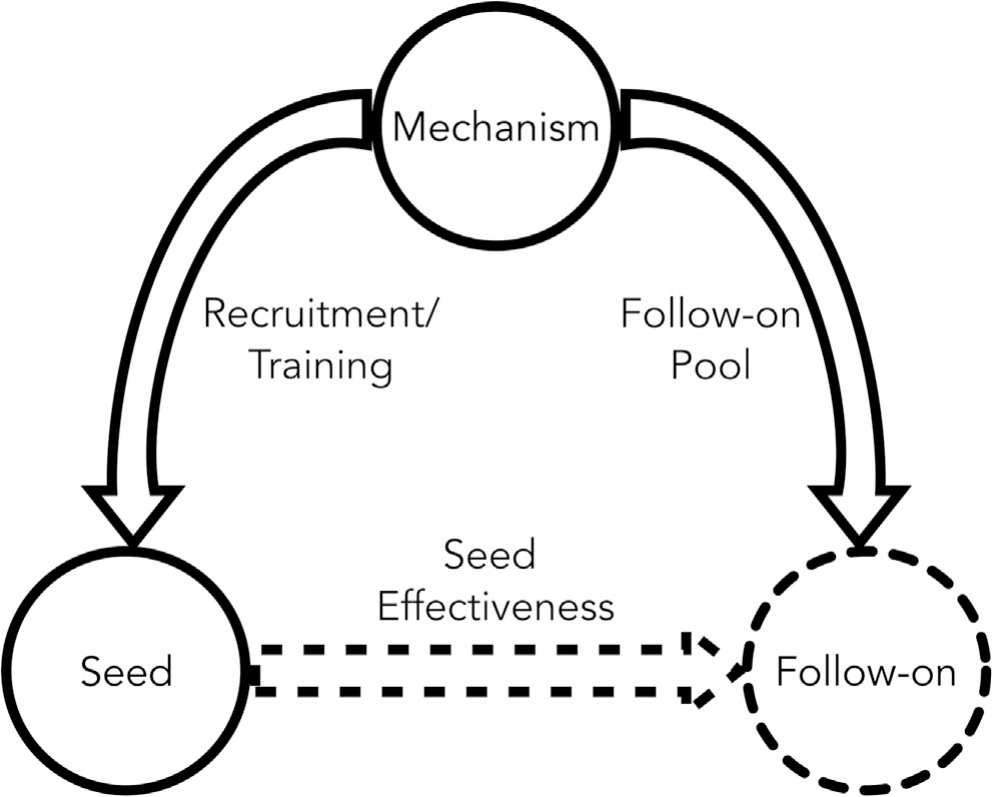
Decisions made in IIP implementation, along with a random variable, drive successful information delivery IIP administrators choose a mechanism, which determines the relationship between seeds and follow-ons. This relationship determines the follow-on pool and informs seed recruitment and training. Thus, the bolded parts of the figure are driven by administrative decisions. The dashed portions—the effectiveness with which seeds are able to engage their follow-on pool and the resulting follow-ons—are unknown to administrators. We call this the *mechanism*: *seeds*–*follow-ons* framework.

Three key design elements are consistent among the diversity of IIPs: **mechanism**, **seeds**, and **follow-ons** (see Figure 2). The **mechanism** for delivering information determines how information sources (seeds) connect to information recipients (follow-ons). For example, some programs are community-focused: local seminars, happy hours, and workshops or community champions rely on spreading information within *already connected* communities or sets of actors (see Table 1). Others, such as the phone ambassador program, create databases to connect potential solar adopters with people who have already adopted solar. Unlike community-focused programs, the phone ambassador program design encourages the creation of *new* connections along which information can travel to potential adopters. **Seeds** are an initial group of individuals recruited by program administrators to spread information. For example, in an intervention using a celebrity advocate, the celebrity is the seed; alternatively, the community champion IIP may use a recruitment drive to find and train community members as seeds to advocate solar PV. The number of seeds recruited and the recruitment and training strategy are administrative decisions in the implementation of the IIP. **Follow-ons** are second-order program beneficiaries that receive information from the seeds. Follow-ons are selected from a “follow-on pool” of individuals who can be reached by the seed, according to the mechanism. The follow-on pool can differ for each seed and across the IIPs. For example, porch talks spread information to community members that walk by the initial contact’s porch, but two different porch talk seeds may live in different communities and interact with different people, thus each has a different follow-on pool. Together these design elements constitute a framework for systematically comparing how IIPs leverage human interactions to influence behaviors. We refer to this as the **mechanism**: **seeds**–**follow-ons** framework.

The **mechanism**: **seeds**–**follow-ons** framework generalizes the diversity of real-world IIPs to facilitate specification and parameterization. Two variables embedded in the framework—*seed count* and *follow-on rate*—establish a common parameter space that describes how information is spread by each IIP. The first parameter, *seed count*, measures the number of seed recruits into the IIP. As a measure of the level of programmatic effort involved in implementing an instance of the IIP, this is expected to scale with administrative cost. For a specified mechanism, the *seed count* parameter serves as the primary lever over which a program designer has control. The second parameter, *follow-on rate*, measures the effectiveness of seeds to reach individuals in their respective follow-on pools. The *follow-on rate* is not a measure over which a program designer has control; instead, it captures an aspect of stochasticity in the ability of a seed to engage with those in their follow-on pool. Reliably effective IIPs are not very sensitive to *follow-on rates*; they generate additional adoptions for a broad range of *follow-on rates*. In addition to the two variables *seed count* and *follow-on rate*, there are other dimensions in the shared parameter space that are not explored here. For example, IIPs may contain different levels of information in their messaging or be differently compelling to motivate follow-ons to update their information levels. We address different levels of information through the training feature described in the Methods section, an exploration of different degrees of motivation is an opportunity for future work.

**Figure 3:**
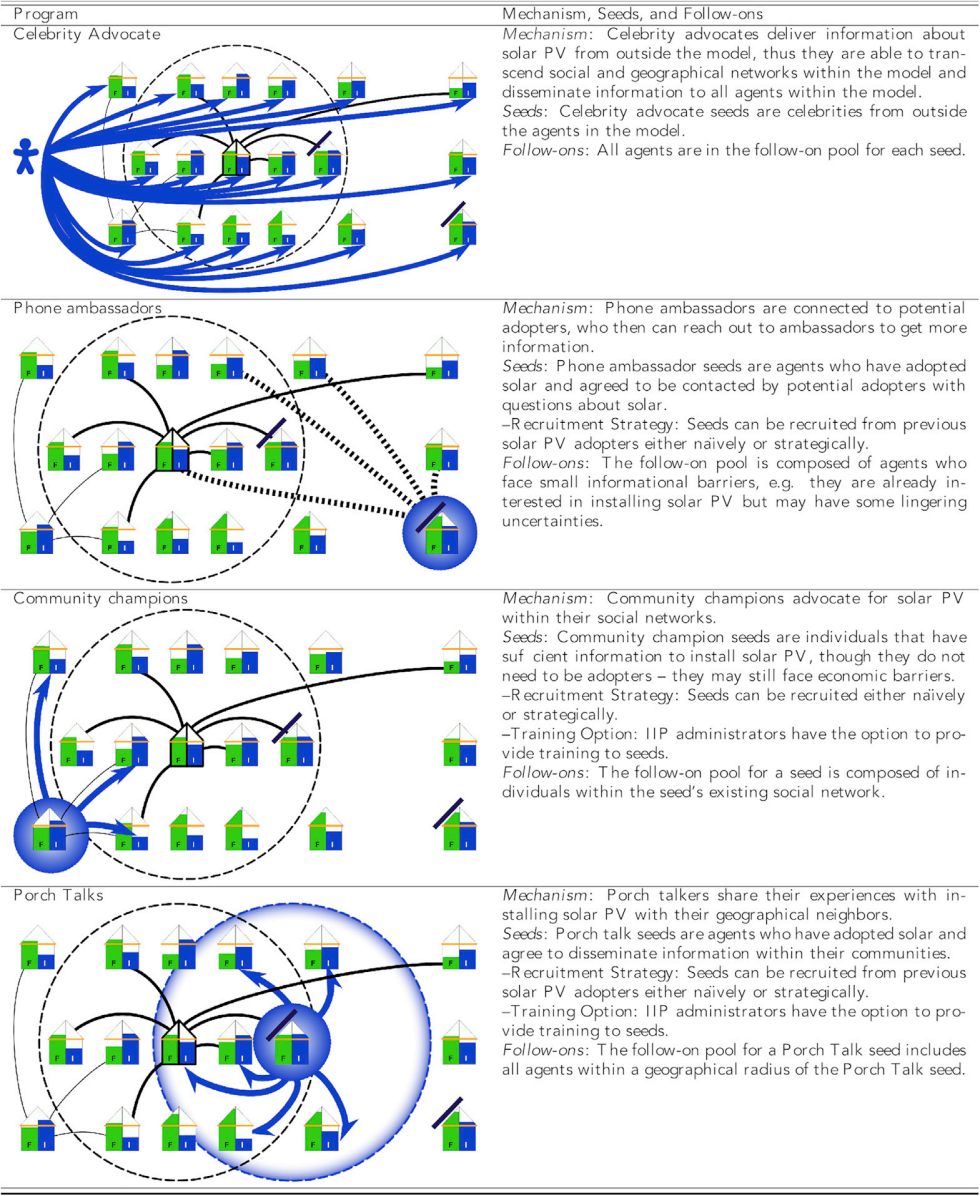
Each of the four archetype IIPs structures interactions between seeds and follow-ons through a different mechanism Each individual has different levels of access to financial (F) and informational (I) resources (F in green and I in blue). Solid black lines indicate social connections, while dotted black lines indicate new social connections; the dashed circle indicates geographic neighbors. Active information channels, show in blue arrows, deliver information from seed to follow-ons in various ways. Notice that there are no active information channels in the phone ambassador mechanism—no exchange of information is enforced in this design. Instead, new and persistent connections are created along which agents may exchange information at any time during the simulation.

Figure 3 presents four archetype IIPs that capture the variation in information exchange design elements found in real-world IIPs, with components of the **mechanism**: **seeds**–**follow-ons** framework identified. To illustrate this framework and its parameter space in an archetype IIP, consider a program administrator tasked with implementing an IIP. The administrator identifies a mechanism, in this example say the “celebrity advocate” IIP, which encodes how a celebrity seed will pass information to its follow-on pool. In the “celebrity advocate” IIP seeds are outside of the target population and the follow-on pool is the entire target population. The IIP administrator chooses a number of celebrity seeds to recruit—the *seed count*. Each celebrity seed then delivers information to the follow-on pool—i.e. the entire population (perhaps through a broadcast advertisement). But only a portion of those in the follow-on pool, according to the *follow-on rate*, will engage with the message. Celebrity advocate seeds are effective to the extent that they can reduce informational barriers for large proportions of the follow-on pool—i.e. if they have a high *follow-on rate*.

In the *seed count*/*follow-on rate* parameter space we operationalize four archetypical IIPs, each with a unique mechanism, that together captures the diversity of the nine real-world solar PV IIPs (see Figure 3). For example, the “community champion” IIP leverages existing social networks that connect seeds to follow-ons, as does the local seminars, happy hours, and workshops IIP (see Table 1). Thus, at a general level, the “community champion” IIP acts as an umbrella for similar programs that use participants’ existing social networks as the mechanism to connect seeds to follow-ons. “Porch talks” spread information within a geographically local community and serve as an archetype for IIPs such as open house and guided tours that rely on a seed’s geographical proximity to spread information to follow-ons. The “celebrity advocate” IIP shares a mechanism with mass media advertising. Each endorsement or ad can have broad geographic or social distribution; there is no requirement that a follow-on be socially connected to a seed. The “phone ambassador” IIP also spreads information outside of existing networks by forging new ties between seeds and follow-ons that were previously unconnected. For relevant IIPs, we explore strategies to enhance the recruitment and training of seeds. Apart from acknowledging that enhancement strategies are likely to incur additional costs, we do not directly account for the increase in administrative overhead. As we show later, in most cases the additional cost of these strategies is not associated with an increase in program effectiveness.

## Modeling

We implement the **mechanism**: **seeds**–**follow-ons** framework in an empirically validated agent-based model (ABM) of individual decision-making as the driver of a diffusion phenomenon (Rai and Robinson, 2015; Robinson and Rai, 2015) that serves as a virtual laboratory to test the effectiveness of archetypical information interventions on the solar PV adoption outcome. The ABM approach is well-suited to the analysis of emergent outcomes—such as behavioral diffusion—that result from interacting, adaptive agents in dynamic contexts (Epstein and Axtell, 1996; Epstein, 2006). A key feature of this ABM is that it explicitly models human decision-making and sheds light on the potential for categorical change in outcomes that result from complex human interactions with relevant interventions—so-called tipping-points or phase shifts. Social tipping-points are thought to be potentially beneficial if they can be systematically identified and strategically used (Lenton, 2020; Otto et al., 2020), and ABM is one tool for identifying them (Castilla-Rho et al., 2017). ABM has an increasingly rich literature as a tool for understanding the diffusion of energy-related technologies, including solar PV (Zhang et al., 2011; Noori and Tatari, 2016; Murakami, 2014; Macal et al., 2014; Rai and Robinson, 2015; Robinson and Rai, 2015).

Despite their growing popularity, a major criticism of ABMs focuses on the abundance of abstract models that lack empirical validation against real-world data, thus limiting the descriptive and explanatory power of the models for meaningful policy evaluation (O’Sullivan et al., 2016). In this study, agents make decisions in a (spatial, social, economic, and policy) context that mirrors the dynamism and complexity of real-world adoption decision-making. The dynamic economic context is driven by real-world values including changing prices, which generally fall over time, and shifting subsidy policies that provide varying rebates and feed-in tariffs. The information context is dynamic and emergent. Agents’ perceptions of local informational norms are initialized by survey data for the relevant population and updated over time through local information exchanges with other agents along edges in a directed small-world network (SWN) of social ties. The thorough incorporation of empirical context and rigorous empirical validation of the baseline model (Rai and Robinson, 2015) adapted here provides a unique opportunity to demonstrate using ABM to model hypothetical outcomes that emerge from an understanding of the interplay between the social, spatial, and economic drivers of individual behavior (Rai and Henry, 2016).

The simulation model operationalizes a dual-threshold simulation model of decision-making that uses a financial activation threshold to capture monetary drivers of adoption and an informational activation threshold (*I*_*threshold*_) to capture the combination of non-monetary factors. When an individual has *both* financial and informational resources above their respective activation thresholds, then the individual adopts the technology. The parsimony of the dual-threshold model is appealing—it is a strength that helps us cleanly capture and distinguish between the differential effects of the programs we simulate.

The empirical context of this ABM—and thus the configuration of this virtual laboratory—is that of the mid-size city in the south of the United States. The diffusion of solar PV, both empirical and simulated, in this context may differ from those in other countries, in more rural or more urban settings, in cities with different climates, or even in similar cities in the same country. However, the strength of an empirically validated virtual laboratory remains the pathway from inputs (e.g. information), through the dual-threshold decision-making model, and to outputs (e.g. solar PV adoption) has been established. Thus, even if the geospatial or temporal pattern of diffusion is tied to this particular empirical context, the results presented here that focus on the relationship between IIPs and subsequent solar PV adoption decisions are generalizable to the extent that individuals process information during their decision-making process similarly. Generalizability to alternative contexts can be enhanced by recalibrating the ABM to a new context; however, doing so is out of scope for the present project.

We do not explicitly account for the spread of information among higher-order beneficiaries (i.e. follow-ons of follow-ons, and so forth). However, once the first-order interactions are actualized in the ABM, higher-order interactions continue to occur as-per-normal inter-agent interactions. All else equal, seeds that have a greater *follow-on rate*—which is to say that they deliver information to a greater percentage of their potential follow-on pool—reach a greater number of agents both directly *and* indirectly through second-hand word-of-mouth; i.e. the ripple effect (Kovacs and Barabasi, 2015; Aral et al., 2013; Hogan et al., 2004). Thus, an IIP that has more first-order interactions is likely to have more higher-order interactions, and the *follow-on rate* adequately proxies all interactions encouraged through an IIP.

We simulate several scenarios for each IIP with different programmatic design elements, e.g. seed counts, recruitment, and training strategies. For IIPs that recruit seeds from the population, seeds are either randomly selected or selected based on high connectivity to other agents—hereafter referred to as “*HighK”* recruiting. HighK recruitment is akin to identifying seeds that a program administrator has reason to believe are highly influential. A large literature has identified more complicated techniques for identifying influential nodes that build upon network centrality and complex contagion theories (Gupta et al., 2018; Kovacs and Barabasi, 2015; Kitsak et al., 2010) that would likely improve the effectiveness of the “HighK” recruitment strategy used here. However, the core of the idea we seek to illustrate is the difference between a naive recruitment strategy (random) and a purposeful recruitment strategy that requires knowing something about the network position of an agent (“HighK”). Although more complicated recruitment strategies were not explicitly explored in this article, they are a potentially important avenue for future study. When an IIP includes training, the level of informational resources that recruited seeds has is “boosted.” Trained seeds are better able to draw their follow-ons above the informational activation threshold. Implementation decisions such as purposeful recruitment strategies and the provision of training add administrative costs to the IIP. For example, recruiting seeds based on individual characteristics—such as “HighK”—increases the administrative burden over a uniformly random recruitment process, likewise providing training to recruited seeds increases the administrative cost of simply accepting seeds as they come. Taken together, these scenarios reflect that to implement an IIP, an administrator must choose from a menu of decision variables available including specifying a mechanism, number of seeds, recruitment strategy, and training. Mechanisms are described in detail in Figure 3.

To simulate an IIP, the baseline model is perturbed once during initialization, i.e. at time *t* = 0. Seeds are recruited according to the recruitment strategy, and the appropriate follow-on pool for each seed is identified. A *follow-on rate* is randomly drawn from *Unif*(0,1), and then each seed’s follow-ons are randomly sampled from the follow-on pool per the *follow-on rate*. For all IIPs except the “phone ambassador,” an information exchange is then enforced between seed and follow-on. For the “phone ambassador” IIP no information exchange is enforced, but the new connection between seed and follow-on persists for the duration of the simulation. As such, for the “phone ambassador” IIP a *follow-on rate* close to one does not imply that each seed interacts with the majority of the follow-on pool, but instead that the majority of the follow-on pool are able to interact with seeds as though they were socially connected. After this, time is advanced to *t* = 1 and the simulation proceeds as originally described (Rai and Robinson, 2015).

### Baseline model overview

The baseline model is lightly adapted from previous work and described at a high level here (additional detail can be found in Rai and Robinson (2015) and Robinson and Rai (2015)). Agents and aspects of the environment (prices, rebates, and so forth) are initialized from empirical data. Agent interactions are structured via a relative agreement algorithm that tracks the level of information (*I*_*i*_) and the uncertainty (*U*_*i*_) about that level. Agent’s uncertainty around their information level determines which interactions with other agents result in an information update and reduction in uncertainty. An update in agent information levels results from interaction with a neighbor whose information level is within the uncertainty bounds of their own information level—information levels outside of these uncertainty bounds are dismissed (Weisbuch et al., 2002; Deffuant et al., 2000, 2002; Meadows and Cliff, 2012).

As agents accumulate information in support of installing solar PV, they become informationally activated when their information levels (*I*_*i*_) surpass a threshold (*I*_*threshold*_). Simultaneously, agents evaluate their financial position relative to the economic context (though this aspect is not the focus of the present work). When agents become both informationally and economically activated, they adopt solar PV. The baseline model is described following the ODD protocol (Grimm et al., 2006, 2010) in SI.

### Informational intervention program simulation

In the ABM, each IIP is conducted once during initialization at time: *t* = 0 and its impacts are simulated over a 6-year period (24 quarters from 2008Q1 to 2013Q4). The adoption outcome is tracked across 1920 independent runs of each IIP with *seed count* and *follow-on rate* parameter values chosen randomly to explore the parameter space (*seed count* ∼ *Unif* (1–500) and *follow-on rate* ∼ *Unif* (0–1)). All other parameters in the model are set to the values fit during the original training and empirical validation procedure (Rai and Robinson, 2015). This allows for a clear comparison between the baseline model and intervention models; the only difference between the baseline model and an intervention model are the parameters for *seed count*, *follow-on rate*, and the intervention itself—i.e. the *mechanism* that connects seeds to follow-ons.

In addition to the two key parameters *seed count* and *follow-on rate*, IIPs can differ by options for seed recruitment method and training.

### Operationalizing informational intervention program mechanisms

*Celebrity advocate* interventions create a number of new agents external to the population of potential adopter agents equal to the value of *seeds*. Each seed (*s*) is assigned a randomly drawn information level (*I*_*s*_ ∼ *Unif* (*I*_*threshold*_, 1) and uncertainty $U_{s}=1-|I|$). Each of these seeds then interacts with agents randomly selected (according to the *follow-on rate*) from the follow-on pool—in this case, the entire population of potential adopters. Interactions follow the typical agent-agent interaction procedures described in the baseline model Rai and Robinson (2015); Robinson and Rai (2015), and happen only once at time *t* = 0.

*Phone ambassador* interventions select a number of seeds from the population of adopter agents (as opposed to potential adopters) equal to the value of *seeds*. *Seeds* may be recruited strategically—a description of strategic recruitment follows. Seeds are then connected to agents from the follow-on pool according to the *follow-on rate*—in this case, the population of potential adopters that are not informationally activated (*I*_*i*_ < *I*_*threshold*_) is ordered by highest *I*_*i*_ to lowest. The social networks of follow-on agents are augmented by adding connections to seeds once at time *t* = 0. Afterward, follow-on agents may or may not interact with seeds following the typical agent-agent interaction procedures described in the baseline model Rai and Robinson (2015); Robinson and Rai (2015)—i.e. no interactions are “forced” but simply allowed to occur as per normal.

*Community champion* interventions select a number of seeds from the population of potential adopter agents who have access to informational resources greater than the threshold necessary for informational activation (*I*_*i*_> = *I*_*threshold*_) equal to the value of *seeds*. *Seeds* may be recruited strategically and/or trained (descriptions of strategic recruitment and training follow). Each seed then interacts with agents randomly selected (according to the *follow-on rate*) from the follow-on pool—in this case, the social network of each specific seed. Interactions follow the typical agent-agent interaction procedures described in the baseline model Rai and Robinson (2015); Robinson and Rai (2015), and happen only once at time *t* = 0.

*Porch talk* interventions select a number of seeds from the population of adopter agents (as opposed to potential adopters) equal to the value of *seeds*. *Seeds* may be recruited strategically and/or trained (descriptions of strategic recruitment and training follow). Each seed then interacts with agents randomly selected (according to the *follow-on rate*) from the follow-on pool—in this case, the potential adopters within a specified geographic radius (the same radius used to create the seed’s SWN). Interactions follow the typical agent-agent interaction procedures described in the baseline model Rai and Robinson (2015); Robinson and Rai (2015), and happen only once at time *t* = 0.

### Seed recruitment

Each IIP experiment has between 1 and 500 agents as *seeds*. Two targeting mechanisms are used for selecting seed agents within the interventions:

*Random recruitment*. Seeds are randomly selected from the pool of potential seed agents. This recruitment strategy represents a minimal cost approach to selecting an IIP participant.

*HighK recruitment*. Seeds that have the highest number of connections (K) to other agents are selected from the pool of potential seed agents. This recruitment strategy represents a more costly approach to selecting an IIP participant based on identifying agents that can potentially influence the most follow-on agents. There are many more advanced recruitment techniques that draw on recent insights in the network science literature (Gupta et al., 2018; Kovacs and Barabasi, 2015; Aral et al., 2013; Kitsak et al., 2010), but all represent the same fundamental tradeoff: they capture the costs/benefits of seeding based on knowing more about agents’ social networks. Implementation of these methods is beyond the scope of this analysis, but suggests an avenue for future research.

### Training

Some IIPs involve training and education of participants (seeds) to be better advocates for solar PV (Noll et al., 2014; Sinclair and Rosoff, 2009; Kowalska-Pyzalska, 2016; Palm, 2016). To simulate the impact of training seed agents in the community champion and Porch Talk interventions we boost *I* and reduce *U* of seed agents per Equations 1 and 2. *I* is boosted as follows:

(Equation 1) $$I_{i,t}=I_{i,0}+\frac{1}{2}\left(1-I_{i,0}\right)\text{,}$$

where *I*_*i*__*,*__*t*_ is the informational level of agent *i* after receiving training and *I*_*i*__*,*__0_ is the informational level of agent *i* before receiving training at time *t* = 0. Uncertainty is reduced per:

(Equation 2) $$U_{i,t}=1-I_{i,t}\text{,}$$

where *U*_*i*__*,*__*t*_ is the uncertainty of agent *i* after receiving training and *I*_*i*__*,*__*t*_ is the informational level of agent *i* after receiving training.

### Comparing the relative value of informational intervention program adoption outcomes

To compare the relative value of interventions, we first evaluate the results of a simple economic intervention whereby a fully subsidized solar PV system is provided to each seed agent at initialization. This simplistic intervention makes the assumption that all seeds that are selected to receive a free solar PV installation accept the system and that those that interact with them interact in the same way they would if the system had not been fully subsidized. In reality, it is likely that some seeds would refuse the installation in which case a subsequent round of seeding may be needed to fully distribute the target number of systems. Interactions between recipients of fully subsidized systems and potential adopters may over-generate or under-generate follow-on installations (for example, if the full subsidy program is still in effect, or if the subsidy program exacerbates issues of institutional trust resulting in hesitancy, respectively). Such an archetypical economic intervention has an easily evaluated economic cost and benefit of:

(Equation 3) ProgramCost($)=(P×N)+O,and

(Equation 4) $$\mathrm{Pr}ogramBenefit\left(\$\right)=P\times N\times \beta_{e}\text{,}$$

where: *N* is the number of *seeds*, *P* is the price of each solar system, *O* is the overhead cost to administer the program, and *β*_*e*_ is an estimate of additional adoptions for each additional economic seed. Rearranging 4, the benefit-per-seed is *P* × *β*_*e*_.

Additional adoptions are calculated by

(Equation 5) $$\hat{A_{i}}=A_{i}-A_{emp}\text{,}$$

where $\hat{A_{i}}$ is additional adoptions, *A*_*i*_ is the amount of adoptions for an informational intervention, and *A*_*emp*_ is the amount of observed empirical adoptions. Empirical adoptions are the actual number of adoptions observed between 2008 and 2013.

We then model an economic intervention in which we randomly select agents and seed them by changing their adoption status to “Adopter.” This is programmatically similar to subsidizing the entire cost of an adoption for seeded agents. Additional adoptions after a simulated economic intervention are calculated per:

(Equation 6) $$\hat{A_{e}}=A_{e}-A_{emp}\text{,}$$

where $\hat{A_{e}}$ is additional adoptions from the economic intervention, *A*_*e*_ is the cumulative adoptions after a simulated economic intervention and *A*_*emp*_ is the observed cumulative empirical adoptions.

We use an OLS regression to estimate the linear impact of additional economic seeds on additional adoptions. To measure the effectiveness of the economic intervention we estimate the regression model in Equation (7):

(Equation 7) $$\hat{A_{e}}=\alpha_{e}+\beta_{e}S_{e}+\epsilon_{e}\text{,}$$

where $\hat{A_{e}}$ is additional adoptions from the economic intervention, *β*_*e*_ is the amount of additional adoptions for one more economic seed, *S*_*e*_ is the amount of economic *seeds*, *α*_*e*_ is an intercept term, and $\epsilon_{e}$ is an error term.

To compare across economic interventions and IIPs, we then use a generalized additive model (GAM) to extract a linear component from the non-linear impact of increases in *seed count* on additional adoptions, while controlling for variation in *follow-on rate* (see Figure S1 in Supplemental Items for the non-linear portion of the relationship.). The estimate *β*_*e*_ of this regression can then be compared to a generalized additive model of the outputs generated by the IIP experiments:

(Equation 8) $$g\left(E\left(\hat{A_{i}}\right)\right)=\alpha_{i}+\beta_{i}S_{i}+f\left(S_{i},F_{i}\right)\text{,}$$

where $\hat{A_{i}}$ is additional adoptions from the informational intervention, α is an intercept term, *β*_*i*_ is the amount of additional adoptions for one more informational seed, *S*_*i*_ is the amount of informational seeds, *F*_*i*_ is the *follow-on rate*, and f() is a tensor smooth of the *seed*, *follow-on rate* surface.

As *β*_*i*_ is the average linear marginal impact of an additional informational seed controlling for *follow-on rate* and *β*_*e*_ is the average marginal impact of an additional economic seed, the relative value of an informational seed compared to an economic seed is given by *β*_*i*_/*β*_*e*_. In the simple economic intervention modeled here, the cost of an economic seed is the cost of an entire subsidized solar system for that seed, thus the ratio *β*_*i*_/*β*_*e*_ provides an estimate of how many solar PV installations each informational seed is worth. Making this relative comparison across interventions requires that implementation be *ceteris paribus*. Each intervention is slightly different, particularly with respect to temporal issues. We establish *ceteris paribus* by making all changes associated with implementing a particular intervention in time *t* = 0. For some IIPs this involves instigating information exchanges at time t=0 which precludes temporal reinforcement between seeds and follow-ons, but for the “Phone Ambassador” IIP the network changes made at time *t* = 0 persist allowing for potential temporal reinforcement between seeds and follow-ons. A much more thorough exploration of temporal reinforcement, and of temporal issues more broadly (e.g. recruiting and retiring seeds over time, and so forth) is out of scope for this analysis but is a very promising avenue for future work.

## Results

To implement the **mechanism**: **seeds**–**follow-ons** framework, we use a dual-threshold simulation model of adoption decision-making that resolves economic and informational barriers to adoption at the individual level. An important aspect of the contribution of this research is the use of an extensively empirically validated simulation model (Rai and Robinson, 2015; Robinson and Rai, 2015) as an experimental laboratory, ensuring that the results have interpretive value for solving practical problems (Watts, 2017; Rai and Henry, 2016). The empirical context for these experiments is Austin, Texas, USA, for 24 quarters from Jan. 01, 2008 to Dec. 31, 2013. The population of agents consists of 173,466 actual single-family homes with geolocations, of which 538 had adopted residential solar PV before Jan. 01, 2008. The underlying network along which information exchanges are modeled is estimated in three stages: 1) candidates for social neighbors are established within a geospatial radius of each agent, 2) geospatial candidates are constrained by homophily to the top 5 percent most similar agents by home value, and 3) the constrained set of candidates is augmented by adding random connections drawn from the entire population to introduce small-world network characteristics (Watts and Strogatz, 1998). The first two stages of estimating the social networks establish the localized connections for information exchanges which lay the groundwork for peer effects, which are an important social determinant of solar PV diffusion (Bollinger and Gillingham, 2012; Rai and Robinson, 2013; Noll et al., 2014; Graziano and Gillingham, 2015; Palm, 2016; Rai et al., 2016; Wolske et al., 2017; Barton-Henry et al., 2021). Additional details of the empirical context, the model, and its implementation are provided in the STAR Methods section, Rai and Robinson (2015), and Robinson and Rai (2015).

Note that neither the framework nor subsequent analysis is fundamentally linked to the simulation model or to this particular empirical context *per se*, rather the framework formalizes each IIP in a way that structures operationalization within the simulation—the framework could just as readily be applied directly to real-world IIP implementations. The simulation model provides a theoretically motivated and empirically grounded environment in which to apply and analyze the framework, which can be calibrated to other empirical contexts. Thus, while the application of this framework has empirical roots in a specific behavior (adoption of solar PV) in a specific geographic, temporal, and policy context, the framework itself is generalizable beyond solar PV adoption to other behaviors that include both monetary and non-monetary factors. Furthermore, by discussing our main findings in terms of fundamental network metrics we establish their generalizability beyond the underlying information network instantiated herein.

**Figure 4:**
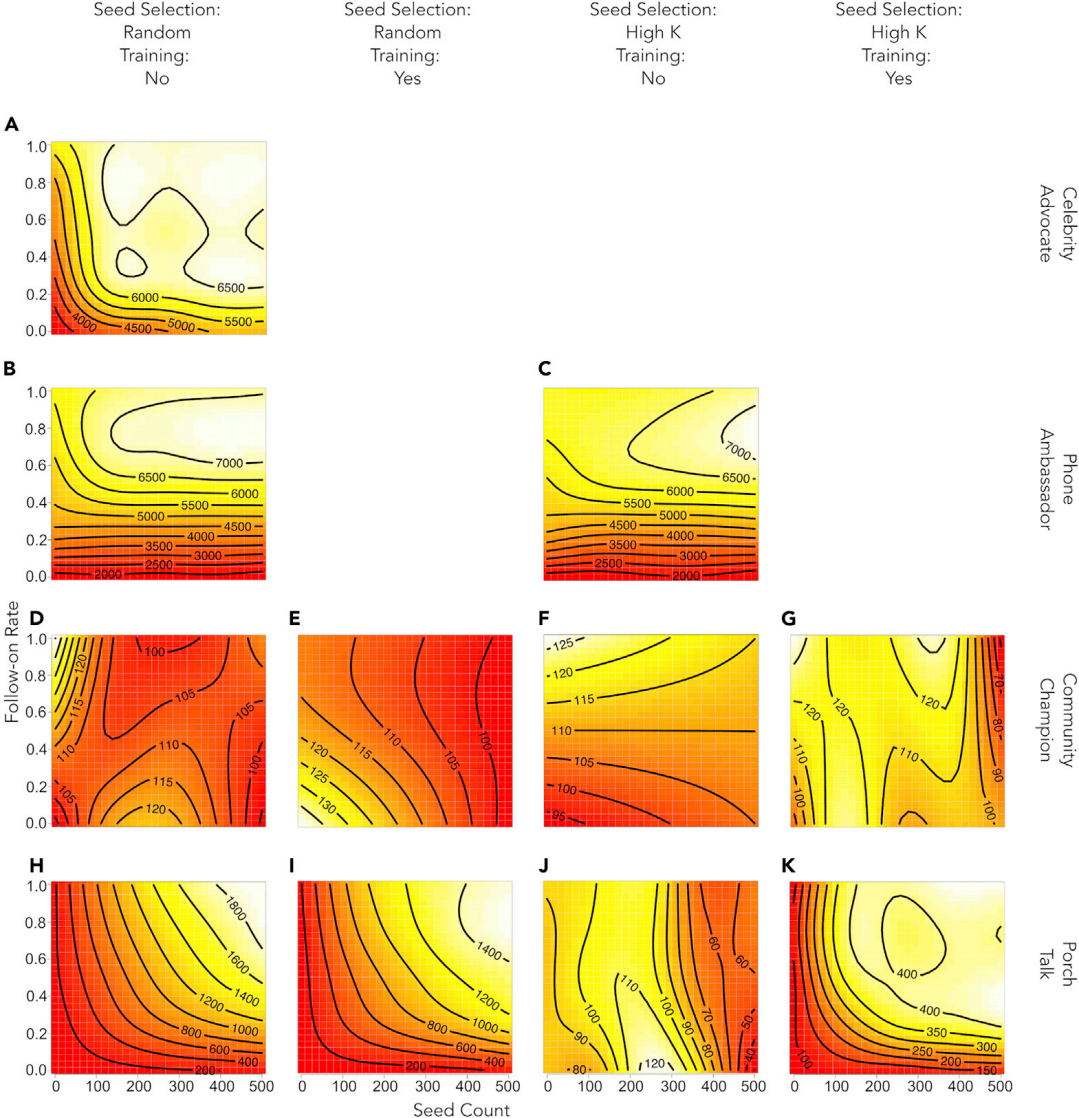
Phase diagrams show the *seeds***–***follow-on rate*–additional adoptions relationship across IIP mechanisms and administrative decisions Depicted here are estimated tensor surfaces illustrating the relationship between the *seed count* (horizontal axis), the *follow-on rate* (vertical axis), and the additional adoptions outcome from low impact (red) through medium impact (yellow) to high impact (white). Strong changes in color indicate shifts in the parameter space that correspond with large, non-linear changes in the outcome. To highlight patterns in adoption responses, the color scale for each pane is calculated independently, i.e. high impact in one pane (e.g. K) may represent only 400 adoptions while the high impact in another (e.g. A) represents 6,500 additional adoptions. This distinction is made clear by the labels of the contours. Panes (A–C) show particularly effective interventions that are capable of generating several thousand additional adoptions. Notice that (A) is more sensitive to increasing *seed count* than (B) and (C). Panes (H, I, K) are less effective than (A–C), but show a similar pattern. (D–G, J), show relatively ineffective interventions that have no discernible pattern in the relationship between *seed count*, *follow-on rate,* and additional adoptions.

**Figure 5:**
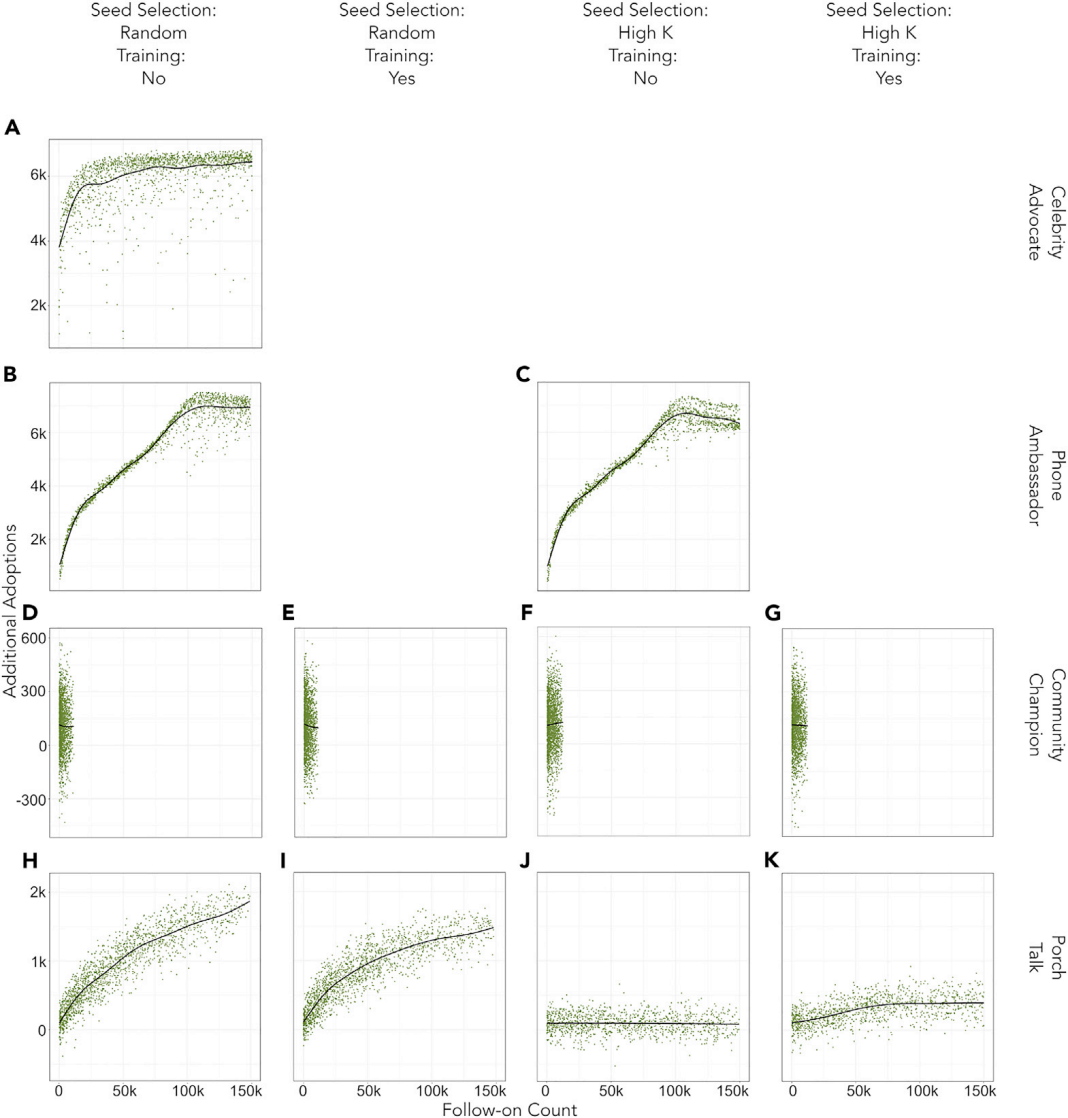
Additional adoptions for each IIP are presented as a function of the *count* of follow-ons (as opposed to the *follow-on rate*) Effective IIPs will demonstrate a strong positive correlation between increasing numbers of follow-ons (information spreading to more people) and additional adoptions, thus demonstrating the value of reaching additional follow-ons. Ineffective IIPs will have flat returns to additional follow-ons. These plots also identify inflections that separate the relationship between additional adoptions and the *follow-on count* into segments, e.g. (A–C) show an inflection point where additional follow-ons cease to have high returns in additional adoptions. This inflection point identifies an information saturation threshold after which economic activation becomes the binding constraint in individual adoption decisions. Note that the relationship between seeds and follow-ons is not shown here—refer to Figure 4.

The key outcome compared across models is *additional* cumulative solar PV adoptions (over the baseline) at the end of the model’s 22 quarter runtime. Two sets of metrics are used to compare adoption outcomes: 1) phase diagrams of additional cumulative adoption outcomes over the *seed count*/*follow-on rate* parameter space, and 2) plots of additional cumulative adoption outcomes versus the actual number of follow-ons reached. Results are presented in Figures 4 and 5.

### Simulation results

The **mechanism**: **seeds**–**follow-ons** framework provides structure to the interpretation of results by connecting input, process, and output. Within a particular IIP mechanism, the number of seeds deployed serves as the primary policy lever: the input. Additional adoptions are the desired output, and the framework allows a glimpse into the process by which inputs generate outputs—a process outside the control of IIP implementers. The *follow-on rate,* therefore, reflects an aspect of uncertainty associated with the process by which a particular mechanism turns inputs into outputs. A stable relationship between seeds and additional adoptions across a range of *follow-on rates* suggests that an IIP mechanism could produce reliable results.

Effective IIPs generally show a positive relationship between additional adoptions and follow-ons which is subject to decreasing returns (Figures 4A, 4B, 4C, 4H, and 4I). The dominant feature in some IIPs is that the initial regime of decreasing returns gives way to the regime of flat returns (Figures 4A, 4B, and 4C). These high-level similarities in the relations between additional adoptions and follow-ons suggest patterns in IIP effectiveness.

The effectiveness of the “celebrity advocate” IIP (Figures 4A and 5A) is intuitive even for a small number of seeds. As celebrity seeds have access to the largest potential follow-on pool within the model, each celebrity advocate seed can exert influence on larger populations of follow-ons than seeds in most other IIPs. Recall that the “celebrity advocate” archetype IIP shares a mechanism with mass media advertising. Thus, while it is likely that a real-world implementation of this IIP archetype using actual celebrities would likely use rather few seeds, implementation of this IIP archetype using mailers or advertisements with large numbers of seeds is not unrealistic.

Figure 5A shows a range of follow-on counts between 0 and approximately 20,000—up to an inflection—in which more follow-ons generally succeed in spurring additional adoptions. This suggests that in this setting a “celebrity advocate” IIP reaches a saturation point at ∼20,000 follow-ons after which returns to additional follow-ons for sparking more additional adoptions are flat. In a setting of ∼175k individuals, this could be achieved with a single celebrity advocate capable of attaining a ∼11.4% *follow-on rate*, or some combination of celebrities with lower *follow-on rates*.

For the “phone ambassador” IIP, impacts are not very sensitive to the *seed count* parameter but compound rapidly as phone ambassadors reach additional follow-ons (Figures 4B and 4C). The relationship between follow-on count and additional adoptions is steep and roughly linear (Figures 5B and 5C) across a broad range of follow-on count values (0-∼100,000). Taken together, this suggests that the “phone ambassador” IIP 1) may be generally effective even with low programmatic effort (low *seed count*), and 2) may yield high returns to individual seeds’ ability to engage additional follow-ons. The “celebrity advocate” and “phone ambassador” IIPs achieve their large impacts in a fundamentally similar way: even a single phone ambassador seed can access a large follow-on pool because the intervention is not constrained by existing social networks.

No instance of the “community champion” IIP generated sufficient additional adoptions to overcome the baseline stochasticity in the model (Figures 4D–4G and 5D–5G). Some variants of the “porch talk” IIP fall between the highly effective “celebrity advocate” and “phone ambassador” IIPs and the less effective “community champion” strategies, particularly when seeds are recruited naïvely (Figures 5H and 5I) as opposed to strategically (Figures 5J and 5K). However, in contrast to the “community champion” IIP, training improves the outcome for the “porch talk” IIP with strategic recruitment, although overall effectiveness in generating additional adoptions still remains low (Compare Figures 5J and 5K).

It should be noted that a positive relationship between follow-on and additional adoptions is not strictly necessary for an effective IIP. Without altering the economic context, to achieve high adoptions without high follow-ons while only operating through information diffusion requires that there be greater higher-order impacts without greater second-order impacts (IE, follow-ons). A “late blooming” IIP that generates high levels of new adoption without correspondingly high follow-ons is both possible and valuable. Here, we adhere to the assumption that increasing higher-order impacts are likely proxied by increasing second-order impacts while leaving open the possibility for such a “late-blooming” IIP to exist.

### Comparing informational intervention program outcomes in terms of a simple economic intervention

Table 2 compares the financial value of intervention programs by comparing the return on investment between simulated IIPs and a simple economic intervention (equivalent to a 100% subsidy) with naive recruitment of seeds. Each additional economic seed yields approximately 1.71 (*β*_*e*_) additional adoptions *inclusive*—the one installation that was subsidized and an additional 0.71 installations per seed that are driven by indirect informational activation. These additional 0.71 installations reflect the degree to which a subsidized installation contributes to the informational context and erodes the substantial informational barriers to adoption illustrated in Figure 1. Setting aside differences in administrative overhead costs, the cost of an economic seed is simply the cost of a solar system for that seed. Thus, with the additional adoptions associated with an additional informational seed (*β*_*i*_), the ratio of *β*_*i*_/*β*_*e*_ provides an estimate of how many economic seeds (*S*_*e*_) each informational seed is worth.

**Table 2:** Assessing the value of information interventions

IIP	Selection	Training	β	Relative Value (*β*_*i*_/*β*_*e*_)
Economic	Random	–	1.713∗∗∗	1.0
			(0.021)	
Celebrity advocate	–	–	4.860∗∗∗	2.837
			(0.076)	
Phone ambassador	Random	–	2.858∗∗∗	1.668
			(0.055)	
	HighK	–	2.505∗∗∗	1.462
			(0.048)	
Community champion	Random	Yes	−0.014	−0.008
			(0.021)	
		No	0.006	0.004
			(0.022)	
	HighK	Yes	−0.019	−0.011
			(0.024)	
		No	0.033	0.019
			(0.021)	
Porch talk	Random	Yes	2.042∗∗∗	1.192
			(0.037)	
		No	2.520∗∗∗	1.471
			(0.035)	
	HighK	Yes	0.528∗∗∗	0.308
			(0.031)	
		No	−0.004∗	−0.002
			(0.025)	

*Note*:^*∗*^p < 0.1; ^*∗∗*^p < 0.05; ^*∗∗∗*^p < 0.01.

In addition to the IIPs, we simulate a simple economic intervention wherein we fully subsidize the installation of solar PV. This intervention generates additional installations beyond each subsidized seed through local information exchanges, here 1.7 additional adoptions per seed (note that *β*_*e*_ and *β*_*i*_ are inclusive of the original seeded adoption—IE they retain their interpretation as a slope from the linear model). Combining this measure of additional installations beyond each subsidized seed as baseline return-on-seeds, we can estimate the relative value of a seed in each IIP. Note that in order to compare a single value of return-on-seeds for IIPs we have extracted the linear component of what are, as shown previously, non-linear impacts. An assessment of the linearity of the components is presented in Supplemental Items, Figure S1.

“Celebrity advocate” IIP seeds have the highest value of all seeds and each seed is, on average and accounting for *follow-on rate*, worth 2.84 times an economic seed. This suggests that if the cost to administer a single “celebrity advocate” IIP with an average *follow-on rate* (0.5) was less than 2.84 times the cost of an economic seed (i.e. the cost to fully subsidize an installation), then the benefits of the decision to implement the “celebrity advocate” IIP should be expected to outweigh the costs compared to the economic intervention. Conversely the “community champion” IIP is unlikely to be highly valuable, despite using a more costly seed recruitment strategy and even if more costly training is provided. This highlights the use of establishing the relative effectiveness of various configurations of archetypal IIPs in Table 2 for practitioners who have access to the details of their specific implementation context, including estimates of the cost of seeds and overhead costs. By combining those specifics with these expectations about effectiveness, practitioners can gain insight into the potential relative cost-effectiveness of specific IIP implementations.

## Discussion

Our framework provides insight into the role of two competing themes that are likely to impact the success of real-world IIPs: the strength of weak ties (SOWT) and reinforcement. Weak ties are known to be critical for diffusion when they enable the spread of information over long distances between otherwise disconnected clusters of individuals (Granovetter, 1978). Characteristic path length—the mean degree of separation between two agents—determines the ability of seeds to reach across weak ties to distant follow-ons. When reinforcement is important diffusion requires receiving a signal from multiple contacts (Centola and Macy, 2007). The “extent to which friends of [an agent] are also friends of each other” (Watts and Strogatz, 1998, p. 441), reflected in clustering coefficients, provides reinforcement outside a seed’s immediate neighborhood; low clustering coefficients facilitate broad diffusion, while high clustering coefficients contribute to localization. The λ parameter in the small-world networks (SWN) that connect these agents impacts both characteristic path length and local clustering coefficients (Watts and Strogatz, 1998; Rai and Robinson, 2015). For phenomena that do not require reinforcement, the SOWT argument leverages the rapid decrease in characteristic path length at low values of λ to diffuse widely through an SWN. As clustering coefficients decrease much less rapidly than characteristic path length in response to increasing λ, more rewiring is necessary for widespread diffusion through an SWN when reinforcement is important.

**Figure 6:**
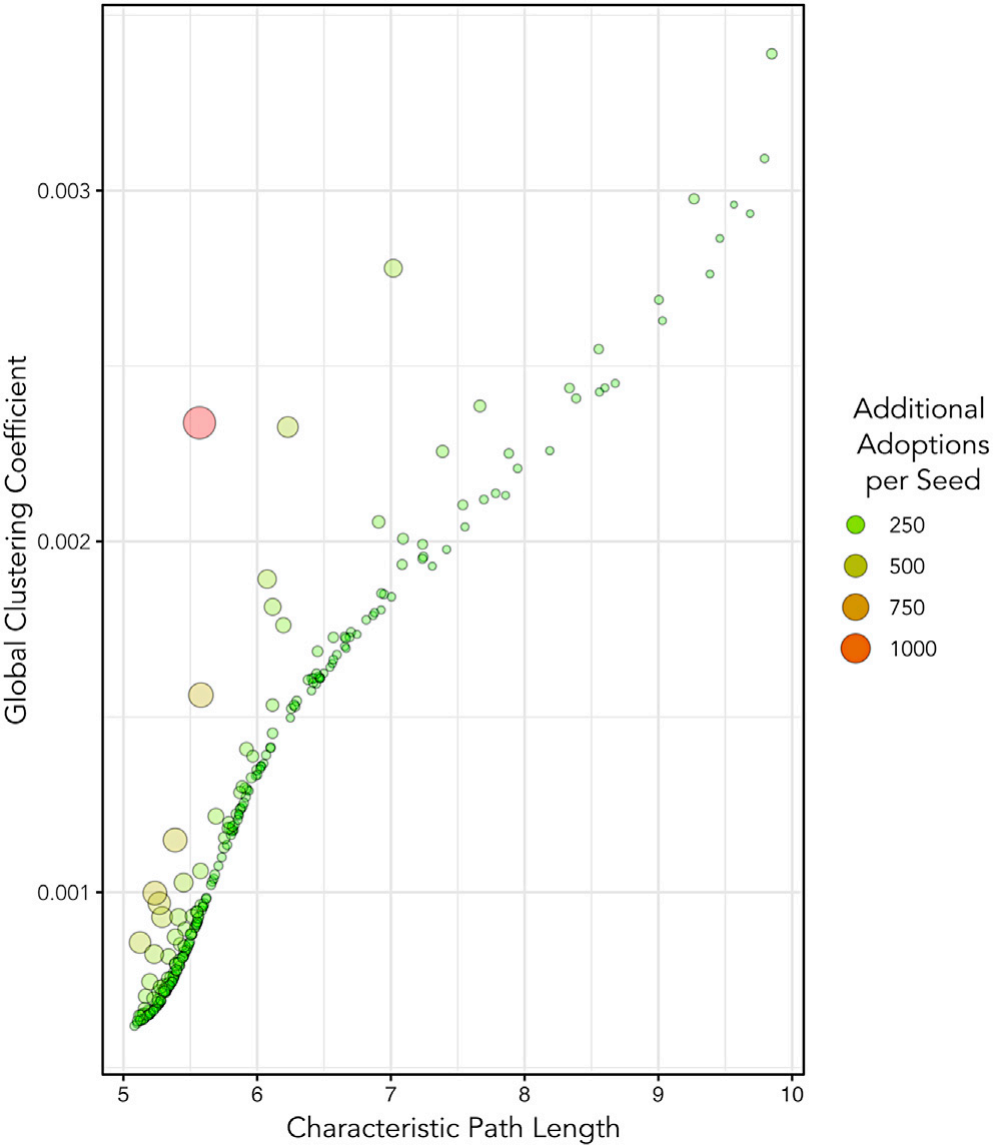
Balancing SOWT and reinforcement drives an effective intervention mechanism The network of *follow-ons* from a random selection of 270 phone ambassador IIPs shows that for any value of characteristic path length, more effective interventions—measured by the ratio of additional adoptions to seeds—manage to introduce more reinforcement through increased clustering in the *follow-on* network.

The *mechanism* plays a critical role in differentiating a successful intervention from a less successful one—the mechanism is much more important than the number of seeds, the strategy for selecting seeds, or whether to train seeds. A mechanism that is a good fit for the target network contributes to the success of an IIP (Proestakis et al., 2018). In this case, interventions that create wide bridges—multiple opportunities for information to travel between distant social groups (e.g. phone ambassador)—balance SOWT and reinforcement and are more effective at encouraging diffusion. Figure 6 illustrates how low characteristic path length and high global clustering coefficients drive the impact of the phone ambassador *mechanism*.

Two examples showcase a mechanism that is a poor fit for the underlying network resulting in an imbalance between SOWT and reinforcement. Comparing Figures 5D–5G to any other pane in Figure 5 shows that even large numbers of “community champion” seeds are unable to engage large numbers of first-order follow-ons—this results in very few weak ties. Many real-world IIPs rely on mechanisms that emphasize reinforcement by strengthening community ties—e.g. community champions, open houses, school events, and group competitions—and do not account for the importance of connecting disparate social groups. Although information exchanged among neighbors is a key driver of solar PV adoption (Rai and Robinson, 2013; Rai et al., 2016; Palm, 2016; Reeves et al., 2017), the nuance here is that agents are exchanging information with exactly the same social neighbors that they could be expected to interact within the absence of the IIP. As the mechanism does not establish the wide bridges necessary for broader diffusion *outside* the seeded cluster, these community-focused IIPs underperform in terms of *additional* installations over the counterfactual expectation with no IIP. Against a backdrop of localized information exchanges that occur naturally, an IIP that encourages creating connections across disparate social groups achieves an impactful balance between SOWT and reinforcement.

A similar imbalance occurs when strategic seed recruitment reduces the effectiveness of the “porch talk” mechanism. Although Figure 5J shows that strategically recruited seeds are able to reach many follow-ons, the most highly connected seeds are likely to be connected to one another in a similar geospatial area (owing to the way social networks are constructed in this model). Additional strategically recruited seeds continually re-seed densely populated regions and re-engage the same follow-on networks that have previously been impacted; high follow-on counts here do not reflect the long-distance aspect of the SOWT argument. Compared to strategically recruited “porch talk” seeds, naïvely recruited seeds have smaller follow-on pools but their follow-on pools overlap much less. Thus, naïvely recruited “porch talk” seeds are more likely to establish wide bridges that enable reinforcement outside of the seeded cluster. In most cases, strategic recruitment is unlikely to be worthwhile, although there are specific instances where it is warranted. For example, adding new connections (edges) to highly connected individuals (as in the “phone ambassador” IIP with strategic recruitment) may encourage the emergence of “hubs” that act as important information conduits; more complicated recruit targeting strategies (Kitsak et al., 2010; Kovacs and Barabasi, 2015) may compound this effect. In practice, fewer nodes with greater connectivity are likely to expose another trade-off: enhanced ability to standardize the message being communicated owing to fewer nodes against the increased workload in communication required of those nodes.

When using a mechanism that is a poor fit for the target network, additional programmatic effort in terms of recruiting additional seeds may be insufficient to achieve increased adoption. In densely connected social groups, information levels trend toward equilibrium over time. In the absence of new information, increasing interactions among seed and follow-on agents do not influence the group equilibrium but only achieves it sooner. Figures 5K and 4K show that introducing additional information to the system (e.g. through training) improves the effectiveness of the “porch talk” IIP with strategic recruitment by altering the level of the group equilibrium. When reinforcement is important, as it is here, new information faces high barriers to saturate densely connected groups. However, new information can rapidly saturate densely connected groups when group members can be convinced to share new information from within.

### Limitations of the study

We caveat these findings by reminding that we do not directly account for the overhead costs of administering any of these interventions. For example, while the “celebrity advocate” IIP provides the best value of all the IIPs, it is also likely that retaining a celebrity and generating the advertisement is the most expensive alternative. We further acknowledge that individual celebrity seeds are likely to have both heterogeneous *follow-on rates* and heterogeneous follow-on pools that may be systematically different based on shared affinities. Including these details are out of the scope of this analysis, but this is another interesting avenue for future work. With these caveats, despite the effectiveness and relative value shown here the “celebrity advocate” IIP may not be the most valuable IIP *in practice*. Recruiting interested individuals to hold porch talks or to serve as phone ambassadors is likely to be less expensive than hiring celebrities, thus “porch talk” or “phone ambassador” IIPs may able to be administered at scale with attractive returns on investment. As noted before, calculations reported in Table 2 offer guidance on the relative cost-effectiveness of the different IIPs. For program designers, the decision to implement an informational or economic intervention is likely influenced by more than just the expected return on programmatic effort; there are likely to be political, social, and distributive equity considerations. Here, we do not advocate for informational interventions over economic interventions but show that informational interventions can, in some contexts, be expected to increase adoption behavior with a reasonably estimated return-on-programmatic effort compared to economic intervention.

## STAR★Methods

### Key resources table

**Table:** 

REAGENT or RESOURCE	SOURCE	IDENTIFIER
**Deposited data**		

Empirical agent descriptions	Rai and Robinson (2015)	https://doi.org/10.1016/j.envsoft.2015.04.014

**Software and algorithms**		

Baseline model code	Rai and Robinson (2015)	https://zenodo.org/badge/latestdoi/225639417
IIP code	This paper	https://zenodo.org/badge/latestdoi/497105850

### Resource availability

#### Lead contact

Further information and requests for resources should be directed to and will be fulfilled by the lead contact, Dr. D. Cale Reeves (d.cale.reeves@gatech.edu).

#### Materials availability

This study did not generate new unique materials.

### Method details

This section describes the reference model based on Rai and Robinson (2015). The model description follows the ODD (Overview, Design concepts, Details) protocol for describing individual- and agent-based models (Grimm et al., 2006, 2010).

#### Purpose

The purpose of this model is to explore residential solar PV diffusion in a dynamic and emergent economic, informational and policy context.

#### Entities, state variables, and scales

The agents in the model are the 173,466 actual single-family residential households in Austin, Texas in mid 2013. Household agents are characterized by the following constant state variables:

• *Index* — A unique identifier for each household ranging from one to n.
• *Coordinates* — Identifies the centroid of the parcel of land for each household.
• *Generation* — Site-specific annual generation estimate (kWh/kW).
• *Insolation* — Site-specific solar insolation (Wh/sq.ft.).
• *Home Value (Hv)* — Proxy for financial resources available ($).
• *Home Size (Ba)* — Footprint of the home, analogous to roof area (sq.ft.).
• *Tree Cover (Tc)* — Site-specific tree coverage (sq.ft).
• *ER* — Access to economic resources ($/kWh/kW).
• *Alters* — Vector of indices identifying agents with which a given agent interacts.

And the following time-varying state variables:

• *Payback* — Calculated payback value ($/kWh/kW).
• *I* — Socially informed attitude about solar PV.
• *U* — Uncertainty about the socially informed attitude.
• *Adopter* — Whether or not the agent has adopted solar PV.

Environmental variables are:

• *Time Step* — Time steps correspond to the interaction opportunities per time period.
• *Time Period* — Time periods correspond to quarters in a year.
• *Price of Solar* — Per unit price of Solar PV ($/W).
• *Rebate* — Local utility rebates for solar PV installation ($/W).
• *ITC* — Federal investment tax credit for solar PV installation ($).
• *Value of Solar* — Production benefit of solar PV generated energy ($/kWh).
• *I Threshold (I*^*∗*^*)* — Global threshold for informational activation.
• *Convergence (μ)* — Coefficient of convergence for the relative agreement algorithm.
• *Radius* — Identifies agents that are geographically neighbors.
• *Homophily (ρ)* — Constrains geographic neighbors to social neighbors.
• *Steps/Period (φ)* — Interaction opportunities per Period.
• *Rewiring(λ)* — Percentage of random connection in agents small-world network.
• *ER scaling intercept(α*_*0*_*)* — An intercept term used to scale ER for comparison to PP.
• *ER scaling slope(α*_*1*_*)* — A slope term used to scale ER for comparison to PP.

Agents near the edge have all their neighbors within the city limits. One time period corresponds to one-quarter, a single simulation is run for 28 quarters over the period from Q1 2008 — Q4 2014.

#### Process overview and scheduling

Each time step, non-adopter agents are tested in random order to see whether they should adopt. The adoption test first updates the agent’s *I* and *U*. Agents with at least one adopter in their network of alters interact with one randomly selected target alter and update their *I* and *U* according to the relative agreement algorithm. The adoption test then tests for two types of activation: informational and economic. Agents with *I* values greater than the *I threshold* are informationally activated; agents with *payback* values less than *ER* are economically activated. Agents that are both informationally activated and economically activated have their *adopter* status set to “adopter” asynchronously.

Each time period, agent’s *payback* values are recalculated with new values for *value of solar* and *rebate*.

#### Design concepts

##### Basic principles

The spatiotemporal pattern of adoption emerges from a dual-threshold process: agent-agent interactions that determine attitudes about solar PV, and agent-environment interactions that determine economic viability. Crossing an informational threshold (“informational activation”) is controlled by the relative agreement algorithm (Weisbuch et al., 2002; Deffuant et al., 2000, 2002; Meadows and Cliff 2012). Crossing the economic threshold (“economic activation”) is undergirded by the Theory of Planned Behavior. Only agents that are both informationally activated and economically activated adopt. This model focuses on being both theoretically grounded and empirically driven.

##### Emergence

The spatiotemporal pattern of adoption, as well as the cumulative adoption curve, are clearly emergent phenomena. Less obvious, and the result of work completed subsequent to the original publication of Rai and Robinson (2015), is the evolution of local informational contexts observed through the spatiotemporal distribution of information especially among non-adopters. Analysis of this “gap” in informational activation is a fruitful approach to studying the exchange of information as an emergent information contagion.

##### Adaptation

Agents adapt by choosing to install solar PV or not. The choice to do so reflects in part an assessment that the benefits of solar PV do not exceed an agent’s individual tolerance for cost. This is not synonymous with benefits greater than costs.

##### Interaction

All agent-agent interactions are direct. Interactions are one-sided; an agent updates based on an interaction with a target agent but the target agent does not update. Communications are not explicitly defined.

##### Stochasticity

Agent networks are small-world networks and are rewired at random during initialization. Target agents for interactions are chosen at random. Models are run in large batches and their outputs, which include stochastic variation as a result of random rewiring and target selection, are aggregated and summarized statistically.

##### Observation

Location and time of adoption are collected for each adoption observed in a single run. A batch of runs is aggregated into a mean cumulative adoption curve and a spatial density of adoption.

#### Initialization

The model is initialized at time *t* = 0 with empirical values for all variables except *index*, *alters*, *I threshold*, *convergence*, *steps/period*, *rewiring*, *ER scaling intercept*, and *ER scaling slope*. Empirical data come from a variety of sources as follows:

• Local utility — Adopter system details, location, installation date, etc.
• Local property tax assessor — Home value, parcel size, land-use, etc
• Local university — Survey responses regarding installation decision
• City government — LIDAR data for household footprint, tree cover
• Council of city governments — LIDAR, service boundaries, surface features, etc
• USGS — Digital elevation data

An agent’s *index* is arbitrarily assigned.

An agent’s network of neighbors (alters) is calculated at initialization by first limiting to the pool of agents located within a radius of 2000 feet. The radius-defined group was culled by 95% to those with the smallest squared difference in home value, the proxy for wealth, to address homophily. Then the parameter *rewiring* percent of the homophily constrained group were rewired to agents chosen at random from the population to form directional small world networks.

The remaining variables *I threshold*, *convergence*, *steps/period*, *rewiring*, *ER intercept*, and *ER slope* are set to the fitted values obtained during model training.

#### Input data

The variables *price of solar*, *rebate*, and *value of solar* are driven by input data. The *price of solar* is modeled as a function of time using a non-parametric LOESS model. Data for these three variables are provided by the local utility.

#### Submodels

There are four submodels: 1) Perceived behavioral control, 2) Agent payback, 3) Information and uncertainty, and the 4) Relative agreement algorithm.

1) Perceived behavioral control is calculated once at initialization for each agent per Equation 9:

(Equation 9) $$ER_{i}=\alpha_{0}+\alpha_{1}\left(insolation_{i}+Hv_{i}-\left(\frac{Tc_{i}}{Ba_{i}}\right)\right)\text{,}$$

where *ER intercept* and *ER slope* are fitted values obtained during training, and *insolation*, *Hv*, *Ba*, and *Tc* are site-specific empirical state variables. *Hv* and the tree-cover ratio (*Tc*/*Ba*) are weighted so that their medians are equal to the median of *insolation*.

2) Agent payback is calculated each quarter for each agent per Equation 10:

(Equation 10) $$PP_{it}=\left(price_{t}-rebate_{t}-\left(price_{t}-rebate_{t}\right)\times ITC_{t}\right)/\left(generation_{i}\times valueofsolar_{t}\right)\text{,}$$

where *price*, *rebate*, *ITC*, and *value of solar* are input data, *rebate* is an environmental empirical state variable, and *generation* is a site-specific empirical state variable.

3) Information levels and uncertainty are estimated once during initiation by combining survey data with spatial regression. I is modeled per Equations 3 and 4.

(Equation 11) $$I_{i}=\beta_{0}+\beta_{1}\;\log\left(Ba_{i}\right)+\beta_{2}\;\log{\left(Ba_{i}\right)}^{2}+\beta_{3}\;\log{\left(Ba_{i}\right)}^{3}+\beta_{4}\left(\frac{Tc_{i}}{Ba_{i}}\right)+\beta_{5}{\left(\frac{Tc_{i}}{Ba_{i}}\right)}^{2}+\beta_{6}{\left(\frac{Tc_{i}}{Ba_{i}}\right)}^{3}+\beta_{7}{\left(\frac{Hv_{i}}{Ba_{i}}\right)}^{2}+\beta_{8}{\left(\frac{Hv_{i}}{Ba_{i}}\right)}^{3}+\epsilon_{i}$$

(Equation 12) $$I_{i}=f\left(\cdot \right)+m_{i}\left(x,y\right)+\epsilon_{i}\left(x,y\right)+\delta_{i}$$

where *I*, *Ba*, *Tc*, and *Hv* are site-specific empirical state variables, *x*, and *y* are location coordinates, f(.) is the linear regression model described in Equation 3, *m*_*i*_(*x*,*y*) is a spatial trend adjustment, $\epsilon_{i}\left(x,y\right)$ is a kriging adjustment (Montero et al., 2015), and *δ*_*i*_ is residual error. *Uncertainty* is initially set to the inverse of *I*.

4) The Relative Agreement algorithm updates *U* and *I* once per time step for agents with at least one adopter among their alters. An agent referred to here as the ego agent interacts with a single target alter and updates *U* and *I* per Equations 13, 14 and 15:

(Equation 13) $$overlap_{eat}=\min\left(I_{et}+U_{et},I_{at}+U_{at}\right)-\max\left(I_{et}+U_{et},I_{at}+U_{at}\right)\text{,}$$

(Equation 14) $$I_{et}=I_{et}+Convergence(\frac{overlap_{eat}}{I_{at}}-1(I_{at}-I_{et})),\text{ if }overlap_{eat}>U_{et\text{,}\text{\&}}=I_{et},\text{ otherwise}$$

(Equation 15) $$\begin{array}{c} U_{et}=U_{et}+Convergence(\frac{overlap_{eat}}{U_{at}}-1(U_{at}-U_{et})),\text{ if }overlap_{eat}>U_{et}\\ ,=U_{et}\text{, otherwise} \end{array}\text{,}$$

where *I*_*et*_ and *U*_*et*_ are time varying state variables for the ego adopter *e* at time *t*, and *I*_*at*_ and *U*_*at*_ are time varying state variables for the target alter adopter *a* at time *t*.

### Quantification and statistical analysis

Statistical analysis was conducted using the R statistical programming language. When results are presented, as in Table 2, asterisks reflect results of two-sided significance tests (*H*0:*β* = 0). The legend is: ^∗^p < 0.1; ^∗∗^p < 0.05; ^∗∗∗^p < 0.01. In all cases n = 1920, and each observation in *n* represents an entire run of the model.

## Data Availability

• Data: The data on which the ABM is trained, and simulation output data reported in this paper will be shared by the lead contact upon request. DOIs are listed in the key resources table. • Code: All original code has been deposited at GitHub (https://github.com/RaiResearchGroup/) and is publicly available as of the date of publication. DOIs are listed in the key resources table. • Other: Any additional information required to reanalyze the data reported in this paper is available from the lead contact upon request.
