# Supplementary material for: Information interventions can increase technology adoption through information network restructuring
Source: iScience. 2022 Jul 20;25(8):104794. doi: 10.1016/j.isci.2022.104794 (PMC9372598; doi:10.1016/j.isci.2022.104794)
Supplement: Document S1. Figure S1 [file mmc1.pdf]

iScience, Volume *25*

Supplemental information

Information interventions can increase technology adoption through information network restructuring

D. Cale Reeves, Matthew Haley, Amara Uyanna, and Varun Rai

Supplemental Items

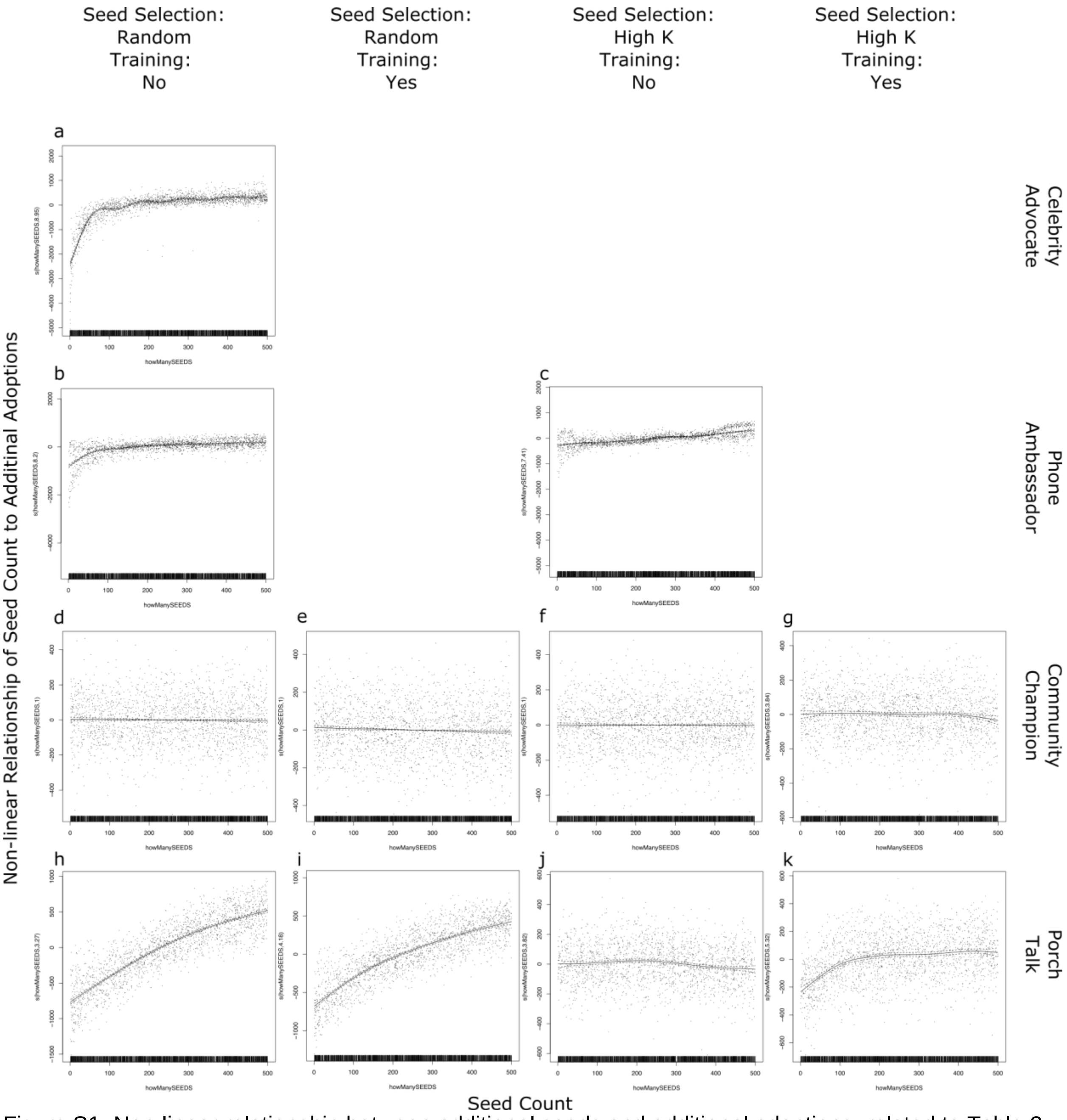

Figure S1: Non-linear relationship between additional seeds and additional adoptions, related to Table 2. When these plots indicate a roughly linear relationship, we have stronger justification for using a linear point estimate in the calculation of economic benefits.
